# Decoding Depression from Different Brain Regions Using Hybrid Machine Learning Methods

**DOI:** 10.3390/bioengineering12050449

**Published:** 2025-04-24

**Authors:** Qi Sang, Chen Chen, Zeguo Shao

**Affiliations:** 1 School of Health Science and Engineering, University of Shanghai for Science and Technology, Shanghai 200093, China; sangqisss01@gmail.com; 2 Center for Medical Research and Innovation, Shanghai Pudong Hospital, Fudan University Pudong Medical Center, Shanghai 201399, China; 3 Human Phenome Institute, Fudan University, Shanghai 201203, China; 4 College of Medical Instruments, Shanghai University of Medicine and Health Sciences, Shanghai 201318, China; 5 Shanghai University of Medical and Health Science Affiliated Zhoupu Hospital, Shanghai 201318, China

**Keywords:** major depressive disorder (MDD), electroencephalogram (EEG), signal processing, machine learning

## Abstract

Depression has become one of the most common mental illnesses, causing severe physical and mental harm. To clarify the impact of brain region segmentation on the detection accuracy of moderate-to-severe major depressive disorder (MDD) and identify the optimal brain region for detecting MDD using electroencephalography (EEG), this study compared eight traditional single-machine learning algorithms with a hybrid machine learning model based on a stacking ensemble technique. The hybrid model employed K-nearest neighbors (KNN), decision tree (DT), and Extreme Gradient Boosting (XGBoost) as base learners and used a DT as the meta-learner. Compared with traditional single methods, the hybrid approach significantly improved detection accuracy by leveraging the strengths of different algorithms. In addition, this study divided the brain regions into the left and right temporal lobes and extracted both linear and nonlinear features to comprehensively capture the complexity and dynamic behavior of EEG signals, enhancing the model’s ability to distinguish features across different brain regions. The experimental results showed that among the eight traditional machine learning methods, the KNN classifier achieved the highest detection accuracy of 96.97% in the left temporal lobe region. In contrast, the stacking hybrid learning model further increased the detection accuracy to 98.07%, significantly outperforming the single models. Moreover, the analysis of the brain region segmentation revealed that the left temporal lobe exhibited higher discriminative power in detecting MDD, highlighting its important role in the neurobiology of depression. This study provides a solid foundation for developing more efficient and portable methods for detecting depression, offering new perspectives and approaches for EEG-based MDD detection, and contributing to the improvement in objectivity and precision in depression diagnosis.

## 1. Introduction

MDD is a mental health condition characterized by persistent sadness, hopelessness, and a loss of interest or pleasure in daily activities [1]. According to estimates from the World Health Organization, more than 300 million people worldwide suffered from depression as of 2023, a widespread condition that affects nearly 1 in 20 individuals globally, making it a leading cause of disability and placing a significant economic burden on healthcare systems worldwide [2]. Currently, depression diagnosis in clinical practice mainly relies on depression scales and clinical symptoms, which are subjective and prone to issues such as missed or misdiagnosis [3]. In recent years, psychiatric research has increasingly focused on methods for capturing and understanding brain activity, such as EEG, functional magnetic resonance imaging (fMRI), and magnetoencephalography (MEG) [4,5,6]. Among these, EEG stands out as a non-invasive neuroimaging technique with significant advantages in depression diagnosis due to its high temporal resolution and ability to monitor brain electrical activity in real time. Compared to other techniques like fMRI, EEG’s non-invasiveness and low cost make it more easily applicable in various clinical settings [7,8,9]. As early as 1994, RoSchke et al. [10] analyzed the sleep EEG signals of depressed patients, extracting the Lyapunov exponent and the correlation dimension D2 to differentiate MDD patients. Subsequent studies have shown that subdividing brain regions improves depression recognition using EEG. For example, in 2021, Jiang et al. [11] divided all the channels into three sections, the frontal lobe, central lobe, temporal lobe, parietal lobe, and occipital lobe, for experimental purposes. The results showed that partitioning significantly improved the accuracy of depression recognition. In 2023, Jianli Yang et al. [12] divided the brain regions into four parts, frontal lobe, temporal lobe, central lobe, and occipital lobe, and conducted a classification analysis of EEG features from each region using Support Vector Machines (SVMs). The results indicated that the temporal lobe was the most prominent region for MDD detection.

In recent years, studies on MDD EEG analysis based on the MPHC dataset (https://figshare.com/articles/dataset/EEG_Data_New-/4244171, accessed on 24 July 2024) have promoted progress in the field to some extent. For instance, in 2023, Jianli Yang et al. [12] achieved a classification accuracy of 92.4% by extracting nonlinear features such as Lempel–Ziv Complexity (LZC) and frequency-domain features such as Power Spectral Density (PSD) and analyzing EEG signals using SVM. Subsequently, another study by Jianli Yang et al. [13] further explored EEG signals under the two paradigms of eyes-closed (EC) and eyes-open (EO) in the resting state. They classified depression using SVM, KNN, and DT classifiers, achieving a maximum accuracy of 94.03% under the different paradigms. In the same year, Nayab Bashir [14] used various traditional machine learning and deep learning methods to screen EEG data for MDD, finding that the KNN model (87.5%) outperformed the Long Short-Term Memory network (LSTM, 83.3%). Furthermore, M Shivcharan [15] employed feature-ranking algorithms to filter important features, compared multiple machine learning algorithms, and evaluated the performance of various models to identify the most suitable solution for real-time depression detection and classification. In 2024, Shalini Mahato et al. [16] also used this dataset, analyzed EEG signals from different brain regions with six different classifiers, identified the temporal lobe as the key region for depression detection, and achieved a maximum classification accuracy of 95.23%. An overview of recent studies shows that an EEG analysis of MDD using the MPHC dataset primarily focuses on two types of methods:

1. The direct application of traditional machine learning and deep learning methods, such as traditional classifiers like KNN and SVM, as well as deep learning models like LSTM [12,13,14,15,16]. Although deep learning has demonstrated potential in pattern recognition and feature extraction, it heavily relies on large-scale, high-quality data and is prone to overfitting issues under relatively limited EEG sample conditions.
2. Most previous studies have adopted a single-classifier strategy, which makes it difficult to fully leverage the advantages and complementarity of different models in feature recognition.

This study proposes a hybrid machine learning approach that integrates multiple traditional machine learning algorithms, significantly improving the classification accuracy of MDD detection while ensuring model stability and generalizability. Compared with single-model or purely deep learning-based strategies, this hybrid approach effectively reduces the risk of overfitting under limited data conditions and captures MDD-related EEG features more comprehensively. Furthermore, this study subdivides the temporal lobe into left and right regions, enhancing the model’s recognition capabilities and clinical applicability through finer brain region partitioning, thereby laying a solid foundation for the future development of more efficient and accessible MDD auxiliary detection systems.

## 2. Materials and Methods

**Figure 1:**
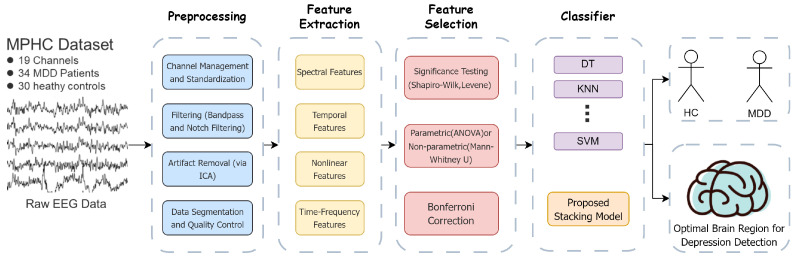
Workflow for EEG-based depression detection.The arrows indicate the sequential data processing pipeline, from raw EEG input to classification.

In this section, we will introduce the overall process framework used in this study for depression detection. Figure 1 illustrates the key steps of this framework: with the patient’s EEG data as input, the data processing flow includes preprocessing, feature extraction, feature selection, and the construction and evaluation of classifiers, ultimately determining the optimal brain region for depression detection.

### 2.1. EEG Dataset

This study utilized the publicly available MPHC dataset [17], which includes data from 34 MDD patients (17 males and 17 females; average age 40.3 ± 12.9 years) and 30 age-matched healthy controls (21 males and 9 females; average age 38.3 ± 15.6 years). All the MDD patients met internationally recognized diagnostic criteria for depression (DSM-IV). The EEG data were recorded using 19 electrode channels with a sampling frequency of 256 Hz and a linked-ear (LE) reference. All the EEG data were subsequently re-referenced to an infinity reference (IR). The recordings included data for the EC and EO resting states, each lasting about five minutes, as well as the TASK conditions. To ensure consistency and quality, all the participants underwent preparation and instructions, such as stabilizing the head and minimizing eye movements, prior to EEG data collection.

### 2.2. Preprocessing

To improve the signal-to-noise ratio and eliminate non-cerebral noise sources, the data preprocessing included channel management, filtering, and artifact removal:

(a) **Channel Preprocessing and Standardization:** Using the MNE library, the irrelevant channels (e.g., ‘EEG A2-A1’, ‘EEG 23A-23R’, and ‘EEG 24A-24R’) were removed. The channel names were standardized following the international 10–20 system. The EEG channels were re-referenced using an average reference method to unify the baseline for waveform analysis.
(b) **Filtering:** EEG signals related to depression typically occur within a 0.5–50 Hz frequency range [18]. An FIR filter was applied to remove low-frequency drift and high-frequency noise, retaining signals within the 0.5–50 Hz range. Additionally, a 50 Hz notch filter was used to suppress power line interference.
(c) **Artifact Removal:** Independent Component Analysis (ICA) was employed to detect and suppress non-cerebral noise sources, such as electrooculography (EOG), electromyography (EMG), and electrocardiography (ECG). Artifacts, particularly those highly correlated with eye movements detected in the frontal channels, were screened and removed based on a threshold criterion to ensure signal purity.

### 2.3. Data Segmentation

**Figure 2:**
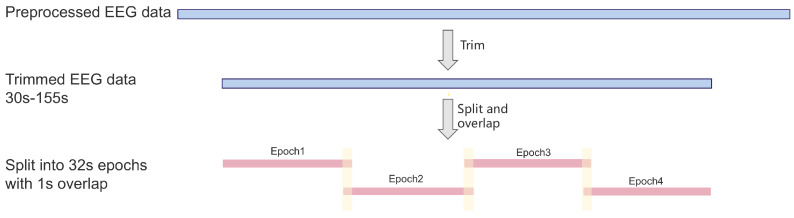
Diagram of data segmentation.

To exclude noise at the start and end of the recordings, only the EEG data from 30 to 155 s were retained for each subject. Each epoch was set to 32 s in length, with a 1-s overlap between adjacent epochs, enhancing data utilization and reducing information loss from window segmentation. Consequently, each subject’s data were divided into four samples with a format of 4 × 19 × 256 (Epoch × Channel × Sample). See Figure 2.

The epochs underwent quality control after frequency filtering and ICA artifact removal. Two threshold criteria were applied to automatically discard low-quality epochs:

**1. Reject Criteria (High Signal)**: Epochs with EEG signal voltages exceeding 100 μV were marked as artifacts and discarded. This threshold filtered out strong artifact signals caused by non-physiological factors, such as eye movements or muscle activity.

**2. Flat Criteria (Low Signal)**: Epochs with EEG signals below 1 μV were marked as invalid due to their lack of physiological significance, often caused by poor sensor connections or other issues.

These thresholds ensured data quality, improving the representativeness of the remaining epochs for feature extraction and classification analysis.

### 2.4. Feature Extraction

**Figure 3:**
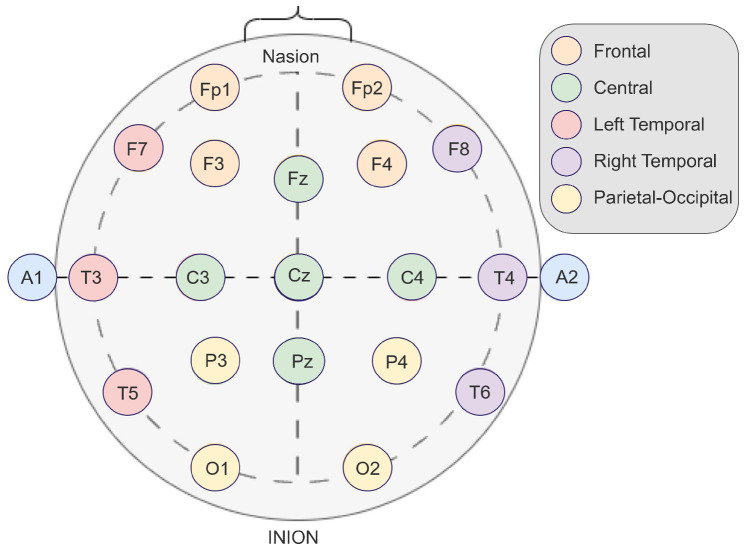
Schematic representation of electrode placement.

The EEG electrodes were positioned according to the international 10–20 system, dividing the brain into five regions: frontal, left temporal, right temporal, parietal–occipital, and central regions (Table 1; Figure 3). Previous studies have shown that different brain regions play distinct roles: for example, the frontal lobe is involved in decision-making, emotional regulation, and social behavior [19], while the temporal lobe plays a key role in long-term memory and emotional state regulation [20].

The basic features of EEG include various waveforms, as well as the characteristics of these waveforms in time and spatial sequences. These waveforms are composed of basic elements, such as frequency, amplitude, and phase. Frequency refers to the number of times a waveform repeats within one second. In this study, the EEG signals are divided into six main frequency bands, ranging from low to high frequencies: Delta (δ; 0.5–4 Hz), Theta (θ; 4–8 Hz), Alpha (α; 8–13 Hz), Beta1 ($\beta_{1}$; 13–21 Hz), Beta2 ($\beta_{2}$; 21–30 Hz), and Gamma (γ; 30–48 Hz).

EEG features related to depression can be categorized into linear and nonlinear features. Linear features typically involve basic statistical analysis and frequency-domain analysis of the signal and can be further divided into frequency-domain, time-domain, and time-frequency features. Nonlinear features reveal the complexity and dynamic nonlinear behavior of the signal. The features included in this study are shown in Table 2.

The features listed above were primarily extracted using the following methods:

**(1)**  **PSD (Power Spectral Density)**
  PSD is an important characteristic used to describe how a signal is distributed in the frequency domain. It reflects the distribution of signal power across various frequencies.
  The power spectra for all the EEG frequency bands were computed using the Fast Fourier Transform (FFT) with a sampling frequency of 256 Hz and a default window length equal to twice the lowest frequency of the band. Initially, the Welch method [21] was applied to calculate the PSD for each electrode across the δ, θ, α, $\beta_{1}$, $\beta_{2}$, and γ bands. To compute the total power in a specific frequency band, we used Simpson’s rule for numerical integration of the PSD. Relative power can also be calculated by dividing the power of a specific frequency band by the total power across the entire spectrum, thereby accounting for the effect of the bandwidth.
  The signal was divided into multiple segments, each processed with a window function and subjected to FFT. The PSD estimates from each segment were averaged to yield the final PSD estimate. This approach not only improves the accuracy of PSD estimation but also facilitates a more precise analysis and interpretation of the frequency-domain characteristics of EEG activity. The specific calculation method is as follows:
  Let the discrete signal *x*, of length *N*, be divided into *K* segments, each containing *M* data points. The starting points of consecutive segments are spaced *R* data points apart, meaning that the overlap between consecutive segments is M−R. The *m*-th segment after windowing is represented as follows:
  (1) $$x_{m}\left(n\right)≜w\left(n\right)x\left(n+mR\right),\;n=0,1,\ldots ,M-1,\;m=0,1,\ldots ,K-1$$
  Here, w(n) represents the window function, and m·R+M=N. The PSD estimate of the *m*-th segment obtained using the periodogram method, $P_{x_{m},M}\left(\omega_{k}\right)$, is given by the following:
  (2) $$P_{x_{m},M}\left(\omega_{k}\right)=\frac{1}{M}{\left|{\mathrm{FFT}}_{N,k}\left(x_{m}\right)\right|}^{2}≜\frac{1}{M}{\left|\sum_{n=0}^{N-1}x_{m}\left(n\right)e^{-j2\pi nk/N}\right|}^{2}$$
  Finally, the PSD estimate of the signal *x*, ${\hat{S}}_{x}^{W}\left(\omega_{k}\right)$, is obtained by averaging the PSD estimates $P_{x_{m},M}\left(\omega_{k}\right)$ of all the segments:
  (3) $${\hat{S}}_{x}^{W}\left(\omega_{k}\right)≜\frac{1}{K}\sum_{m=0}^{K-1}P_{x_{m},M}\left(\omega_{k}\right)$$
**(2)**  **Hurst Exponent**
  The Hurst Exponent (*H*) is a measure of the long-range dependence and persistence of a time series. It assesses the persistence or anti-persistence of the signal by analyzing its fluctuation behavior.
  For a given time series $x=[x_{1},x_{2},\ldots ,x_{N}]$, the cumulative deviation sequence Y(t) is constructed as follows:
  (4) $$Y\left(t\right)=\sum_{i=1}^{t}\left(x_{i}-\bar{x}\right)$$
  where $\bar{x}$ is the mean of the time series. For the cumulative deviation sequence Y(t), the range R(t) and the standard deviation S(t) are calculated. The normalized range R/S is given by (R/S)(n), and the Hurst Exponent is calculated by fitting the relationship between the normalized range and the time length on a logarithmic scale:
  (5) H:log(R/S)∼H·log(n)
**(3)**  **Sample Entropy**
  Sample entropy is a method for measuring the complexity and uncertainty of a time series. It evaluates the signal’s complexity by calculating the probability of pattern occurrence within the signal. The larger the sample entropy, the more complex the signal, and conversely, the smaller the sample entropy, the simpler the signal.
  The specific calculation method is as follows: For a given time series $x=[x_{1},x_{2},\ldots ,x_{N}]$, a pattern vector of length *m* is constructed as $X_{i}=\left[x_{i},x_{i+1},\ldots ,x_{i+m-1}\right]$. For each pattern vector $X_{i}$, the distance to other pattern vectors is calculated (usually the maximum distance), and the number of pattern vectors with a distance less than *r* is counted. Sample entropy is defined as follows:
  (6) $$SE\left(m,r,N\right)=-\ln\left(\frac{A}{B}\right)$$
  where *A* and *B* are the number of matching pattern vectors, respectively.
**(4)**  **Higuchi Fractal Dimension**
  The Higuchi fractal dimension is a method used to describe the complexity and self-similarity of a time series. It evaluates the complexity of the signal by calculating its dimension at different scales.
  For a given time series $x=[x_{1},x_{2},\ldots ,x_{N}]$, a new time series $x_{m}^{k}$ is constructed using different scale factors *k*, where *m* is the initial point and *k* is the scale factor. For each new time series $x_{m}^{k}$, the length L(m,k) is calculated. For a given scale factor *k*, the average length L(k) of all new series is calculated. The fractal dimension is estimated by fitting the relationship between the average length and scale factor on a logarithmic scale, as follows:
  (7) D:log(L(k))∼D·log(k)

### 2.5. Feature Selection

In machine learning and statistical pattern recognition tasks, the features extracted from datasets may contain redundant or irrelevant information. These features not only increase computational burden but may also lead to model overfitting, thereby affecting predictive performance. Therefore, feature selection is a crucial technique that reduces the dimensionality of the feature space by selecting the most effective subset of features, thus optimizing the classification performance of the model.

After feature extraction in Section 2.4, we obtained 95 features (all features) and then proceeded with significance testing to evaluate the relationship between each feature and the target variable (e.g., depression status). Significance testing, a common method in statistical hypothesis testing, helps researchers determine whether differences between groups are statistically meaningful. This testing can be divided into parametric and non-parametric methods, with the choice depending on the data’s distribution characteristics and sample size. Parametric tests typically require data to meet the normality and homogeneity of variance assumptions, while non-parametric tests do not have specific distribution requirements, making them more widely applicable.

**Figure 4:**
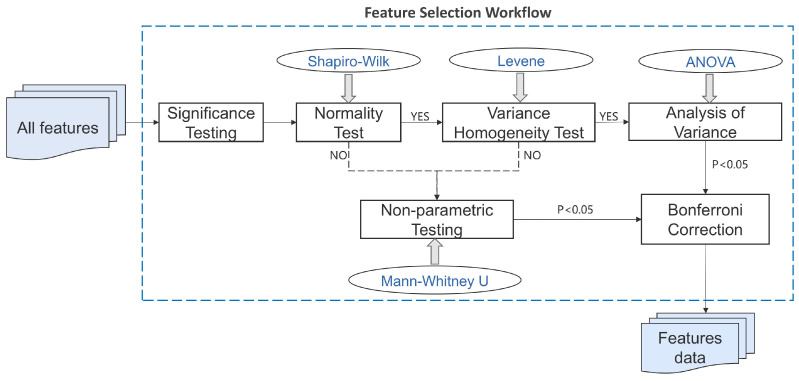
Feature selection workflow.

In this study, normality testing was performed for each feature using the Shapiro–Wilk test to assess whether the data followed a normal distribution. This was followed by Levene’s test to check for homogeneity of variance between groups. The results of these tests determined whether parametric (ANOVA) or non-parametric (Mann–Whitney U) methods should be applied for further analysis. Based on the outcomes of the normality and variance tests, significance testing was performed on each feature. If a feature showed significant differences between groups, it was considered potentially useful for distinguishing healthy individuals from patients. All *p*-values for significance were adjusted using the Bonferroni correction to control the false discovery rate in multiple comparisons. See Figure 4.

**Figure 5:**
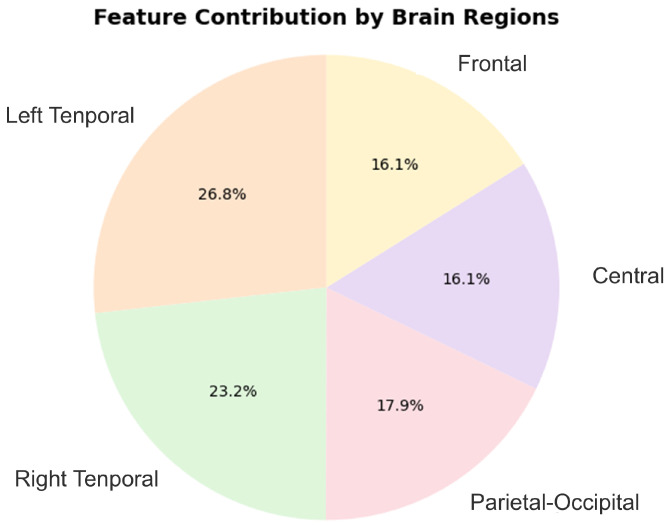
Contributions of features by brain region.

By applying these statistical tests, we selected features that were significantly correlated with depression status from the candidate features. The number of features was reduced from 95 to 56 valid features. This not only alleviated the computational burden on the model but also significantly improved its explanatory power and prediction accuracy in practical applications. Initially, the dataset contained 95 features, which were evenly distributed across the five brain regions (frontal, central, left temporal, right temporal, and parietal–occipital), meaning each region contributed approximately 19 features. See Figure 5. Among the remaining 56 valid features, 26.8% came from the left temporal lobe, 23.2% from the right temporal lobe, 17.9% from the parietal–occipital region, and 16.1% each from the frontal and parietal lobes. These findings suggest that the temporal lobes, particularly the left and right temporal lobes, play a crucial role in depression detection, contributing the largest proportion of features. This indicates that the temporal lobes are key to emotional regulation and cognitive function. While the parietal–occipital, frontal, and parietal regions also contribute, their role is comparatively smaller. This highlights that, although depression involves multiple brain regions, the temporal lobes are particularly sensitive for identifying depressive states, further supporting their central role in depression research.

### 2.6. Classifiers

This study employed various classifiers to identify patients with depression. These classifiers encompass a wide range of model characteristics, including linear and nonlinear models, distance-based classifiers, tree-structured ensemble models, and neural network models, as summarized in Table 3.

In further experiments, we applied the stacking ensemble learning strategy. Stacking is a classical method in ensemble learning that integrates the predictions of multiple base learners into a secondary model, known as the meta-learner. By leveraging the strengths of individual models and compensating for their weaknesses, stacking constructs an ensemble model with enhanced performance and improved generalization capabilities.

**Figure 6:**
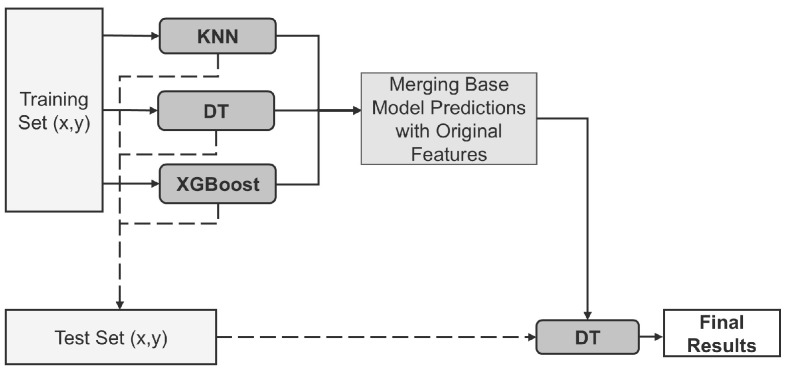
Diagram of the stacking structure.

In our implementation, we selected KNN, DT, and XGBoost as the base learners, with a DT serving as the meta-learner. Figure 6 illustrates the detailed stacking process: the base learners generate predictions for the training set samples, and these predictions are combined with the original features to create a new training set. This new training set is then used to train the meta-learner. Finally, the meta-learner predicts the test set samples, providing the final output.

### 2.7. Evaluation Metrics

During the evaluation of model performance, we employed a set of common evaluation metrics, including the following:

- **Accuracy**
  (8) $$\mathrm{Accuracy}=\frac{TP+TN}{TP+TN+FP+FN}$$
- **Recall**
  (9) $$\mathrm{Recall}=\frac{TP}{TP+FN}$$
- **F1 Score**
  (10) $$\mathrm{F1\_Score}=2\cdot \frac{\mathrm{precision}\cdot \mathrm{recall}}{\mathrm{precision}+\mathrm{recall}}$$
- **Area Under the Curve (AUC)**
  The ROC curve is a commonly used tool for evaluating the performance of predictive models, revealing the continuous relationship between sensitivity (recall) and specificity (1–false positive rate). The AUC, the area under the ROC curve, is a key metric for assessing the performance of binary classification models. The AUC measures a model’s ability to rank positive samples ahead of negative samples, with values ranging from 0.5 (no discrimination ability) to 1 (perfect discrimination ability).
  Definitions:
  **True Positive (TP)**: The number of MDD samples correctly classified as MDD by the proposed method.
  **False Positive (FP)**: The number of healthy controls incorrectly classified as MDD by the proposed method.
  **True Negative (TN)**: The number of healthy controls correctly classified as healthy controls by the proposed method.
  **False Negative (FN)**: The number of MDD samples incorrectly classified as healthy controls by the proposed method.

In classifier performance evaluation, increases in accuracy, recall, F1 score, and AUC values all indicate improved classification efficacy. Accuracy quantifies the proportion of samples correctly labeled by the classifier; recall describes the proportion of actual positive cases identified by the model; and the F1 score, the harmonic mean of precision and recall, is particularly suitable for imbalanced datasets. Together, these metrics provide a comprehensive assessment of model performance, aiding in the identification and optimization of classification algorithms for practical applications.

## 3. Results

### 3.1. Classifier Results

The five-fold cross-validation method was employed during model training. The stacking model achieved the best performance across the metrics of accuracy, recall, and F1 score. The recall of the stacking model reached 98.06%, slightly lower than the highest recall of 99.81% achieved by KNN. Overall, the stacking model demonstrated the best comprehensive performance. The detailed evaluation metrics are shown in Table 4, and the best parameters of each model are presented in Table 5.

### 3.2. Brain Region Detection Results with the Proposed Hybrid Model

To evaluate the contribution of different brain regions to depression detection, we performed a detection analysis on a single brain region under the EC condition using the proposed hybrid model. The goal of this analysis was to assess how different brain regions contribute to the identification of depression-related EEG patterns. By analyzing the performance of individual brain regions, we aimed to identify the brain regions that provide the most significant features for accurate depression detection. The results revealed that the left temporal lobe exhibited the best performance in depression detection, with all the performance metrics reaching their highest values. This highlights the critical role of the left temporal lobe in distinguishing depression-related EEG signals. Detailed results are presented in Table 6.

### 3.3. Analysis of Frequency Band Features and Brain Region Features

**Figure 7:**
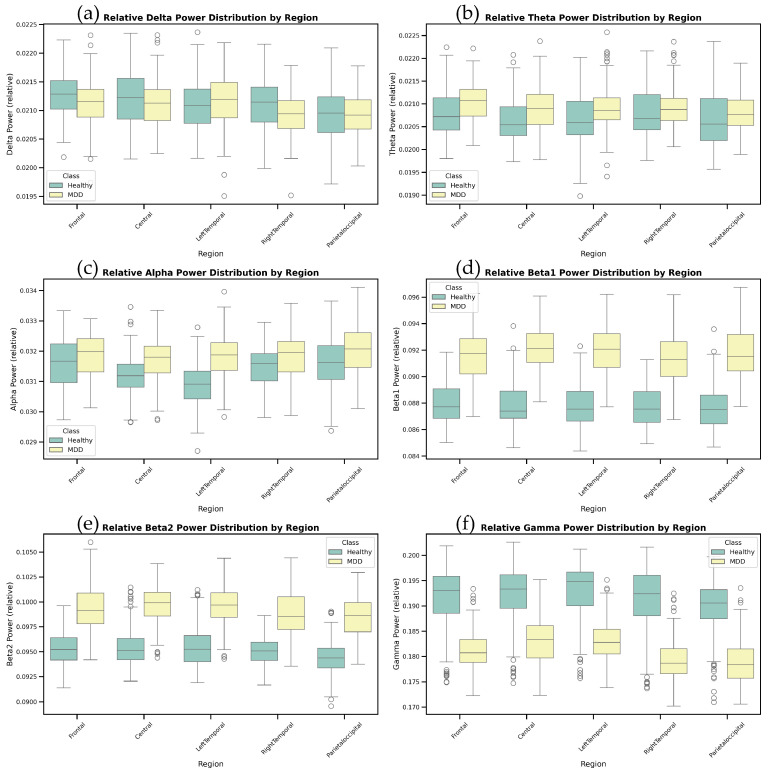
Statistical analysis results of six frequency bands across different brain regions: (**a**) Delta waves; (**b**) Theta waves; (**c**) Alpha waves; (**d**) Beta1 waves; (**e**) Beta2 waves; and (**f**) Gamma waves.

The relative power distribution of six specific frequency bands—Delta, Theta, Alpha, Beta1, Beta2, and Gamma—across brain regions including the prefrontal lobe, left temporal lobe, right temporal lobe, and parieto-occipital lobe is shown in Figure 7.

In this study, the EEG signals were divided into six primary frequency bands, ranging from low to high frequencies, Delta (δ; 0.5–4 Hz), Theta (θ; 4–8 Hz), Alpha (α; 8–13 Hz), Beta1 ($\beta_{1}$; 13–21 Hz), Beta2 ($\beta_{2}$; 21–30 Hz), and Gamma (γ; 30–48 Hz), with each band specifically associated with distinct neuropathological mechanisms in depression (detailed in Section 4.2). In the box plots, the horizontal axis represents brain regions, and the vertical axis indicates relative power values. Different colors in the plots represent different participant groups, with green for healthy participants and yellow for patients with depression. Each box plot illustrates the power distribution in a specific frequency band for all the participants across the different brain regions, providing insights into the differences in EEG activity between the two groups.

Each brain region is represented by an independent box, with fixed width, indicating that the number of samples in each category is equal. The horizontal line within each box represents the median, while the upper and lower boundaries of the box correspond to the third quartile (Q3) and the first quartile (Q1), respectively, providing insight into the central tendency and dispersion of the data. The small circles in the plot represent outliers, which are data points that deviate significantly from the main dataset.

Through these box plots, we can compare the power differences between the healthy group and the depression group across different brain regions. Especially in the Theta, Beta, and Gamma frequency bands, we observed that the depression group exhibited significantly different power distributions in certain brain regions (such as the right temporal lobe and parieto-occipital lobe) compared to the healthy group. These differences suggest potential abnormalities in emotional regulation and cognitive processing in patients with depression, which helps to further understand the relationship between brain regions and depression, providing important insights for EEG-based depression detection.

## 4. Discussion

### 4.1. Classifier Comparison and Analysis

The use of a stacking model ensures a more objective decision-making process. By combining the predictions from multiple diverse models, the stacking approach reduces potential bias arising from any single classifier. Each base model contributes based on its own mechanism of learning, and this diversity leads to more balanced and objective predictions, minimizing the risk of overfitting to specific patterns in the data.

In our experiments, we constructed a custom classification model based on stacking, which significantly improved performance by leveraging the strengths of multiple base classifiers. We selected KNN, DT, and XGBoost as the base learners to harness their varied adaptability to depression EEG features. Specifically, KNN focuses on measuring similarity between data points, DT models decision rules for classification, and XGBoost enhances prediction accuracy by iteratively correcting errors from previous models. These complementary perspectives enable the uncovering of potential patterns within the data.

After training on the initial dataset, the base learners generated a new feature set—consisting of their predictions—which was then concatenated with the original features and fed to a meta-learner. By combining both the base learners’ outputs and the raw features, the meta-learner can leverage each model’s strengths while still retaining essential information from the original data, thereby refining the final predictions.

To further optimize classification performance, we employed a DT as the meta-learner to integrate the predictions of the base learners, enhancing the overall generalization ability of the model. The choice of DT is particularly beneficial due to its interpretability and its effectiveness at handling complex, nonlinear decision boundaries.

Among the traditional models alone, KNN achieved the highest classification accuracy (96.97%), highlighting its strong adaptability to depression EEG features. However, the stacking model, which combines the advantages of all three base classifiers, not only improved accuracy but also demonstrated superior performance in recall, F1 score, and ROC AUC. This improvement stems from the stacking method’s ability to draw on the complementary strengths of different base models, thereby achieving better generalization and more reliable predictions.

This custom classification model exhibited excellent performance on the validation set, further confirming the potential and advantages of the stacking approach for classifying depression EEG signals.

### 4.2. Frequency Band Feature Analysis

(a) Delta Waves

Delta waves are closely associated with deep sleep, restorative rest, and physical recovery processes. These waves are typically most prominent during the initial stages of sleep and in deep sleep, reflecting the body’s rest and recovery state. The results indicate minimal differences in the distribution of Delta waves between the two groups, although slight differences were observed in the parieto-occipital region.

(b) Theta Waves

Theta waves are strongly linked to memory processing, emotional responses, and internal attentional processes. They are more active during deep relaxation, meditative states, or specific emotional states. Our results show that the relative power of Theta waves is generally higher in most brain regions of patients with depression compared to the healthy group, with particularly notable differences in the occipital and right occipital regions. This may indicate differences in emotional processing and attentional regulation in these brain areas for patients with depression. However, compared to other frequency bands, the differences in Theta waves between the healthy and depression groups were not statistically significant.

(c) Alpha Waves

Alpha waves are primarily associated with relaxation, meditation, emotional regulation, attention control, and the ability to inhibit irrelevant stimuli. These waves are typically enhanced in quiet and relaxed states. Our results indicate that healthy participants have lower relative Alpha wave power in most brain regions compared to patients with depression, aligning with the findings of Huang et al. [22].

(d) Beta1 and Beta2 Waves

Beta waves are linked to alertness, anxiety, cognitive load, decision-making processes, and active thought, playing a significant role in emotional control and social interaction. They are typically heightened during intense focus or complex problem-solving. Our findings indicate that patients with depression have higher relative Beta wave power in most brain regions, with Beta2 waves showing particularly significant increases in the frontal region.

(e) Gamma Waves

Gamma waves are associated with advanced cognitive functions, information processing, visual perception, image recognition, and heightened states of consciousness. Their activity is believed to be linked to memory formation and attention integration, particularly during complex cognitive tasks. The results show significant differences in Gamma waves in the parieto-occipital and right temporal regions, where patients with depression typically exhibit lower relative Gamma wave power.

### 4.3. Brain Region Feature Analysis

The results of brain region feature detection using our proposed hybrid machine learning method (based on the stacking model) showed that the left temporal lobe performed best in detecting MDD, with all the metrics reaching their highest values. This may be related to the function of the temporal lobe and its key role in emotional and cognitive processing. The temporal lobe is primarily responsible for language, emotion, and memory processing and is a core region for emotional regulation in the brain. Depression is often accompanied by emotional dysregulation and cognitive dysfunction, so the temporal lobe is particularly sensitive in reflecting these changes [20,23,24].

The left temporal lobe is especially important in emotional regulation, language comprehension, and self-recognition. Therefore, in patients with depression, neural activity in the left temporal lobe may be more easily affected, making the related EEG features more pronounced, thus improving detection accuracy, recall rate, and other metrics. The right temporal lobe, as an auxiliary region, also plays an important role. However, compared to the left temporal lobe, the left lobe shows higher detection ability due to its more direct involvement in emotional and cognitive functions. Moreover, the temporal lobe has relatively fewer channels than other brain regions, which may reduce data noise, thus aiding in more accurate extraction of depression-related features and enhancing model classification performance.

From the median plots, we can see the following:

Differences in the Temporal Lobe: Significant differences between the left and right temporal lobes in the Beta and Gamma bands suggest that these regions may play different roles in emotional processing and cognitive dysfunction in MDD. Particularly, the right temporal lobe may have a more prominent role in processing negative emotions.

High-frequency Activity in the Frontal and Central Regions: Power differences in the Beta and Gamma bands suggest that high-frequency activity in these regions may be closely associated with cognitive dysfunction in MDD. These regions’ activity could be key indicators for MDD detection.

Visual Processing in the Parietal–Occipital Regions: In MDD patients, the increase in Alpha waves and decrease in Beta/Gamma waves in the parietal–occipital regions may reflect abnormalities in visual processing and spatial cognition.

Despite the achievements of this study, there are still several limitations that warrant further attention. To address these limitations, we propose corresponding future work to improve and expand upon the current findings. Firstly, the dataset used in this study is relatively small, and the limited number of samples may affect the generalizability and robustness of the conclusions. Future work will focus on expanding the dataset by including a larger number of samples from diverse populations and geographic regions, which will enhance the external validity of the findings and provide a more comprehensive understanding of depression detection across various demographic groups. Secondly, the dataset lacks critical information on patients’ age, gender, and other medical background details. This limitation prevents an in-depth analysis of how these factors influence depression detection and hinders the exploration of individual differences. Future work will incorporate richer patient metadata, such as demographic information, medical history, and lifestyle details, to enable a more nuanced analysis. This will also facilitate the development of personalized detection models tailored to different subgroups. In addition, this study employs standard preprocessing and feature extraction techniques, but there is room for improvement in EEG signal handling, particularly in noise reduction and artifact removal. Future work will explore advanced signal processing methods, such as adaptive filtering and deep learning-based denoising approaches, to further refine EEG data quality and optimize feature extraction. Lastly, this study focuses on single-modality EEG analysis, which may limit its ability to capture the multifaceted nature of depression. Future work will investigate multimodal approaches by integrating EEG data with other biomarkers, such as neuroimaging, genetic data, or physiological signals (e.g., heart rate variability). This multimodal joint analysis is expected to provide a more comprehensive and robust understanding of depression and further enhance classification performance.

In conclusion, while this study has provided valuable insights into depression detection using EEG data, addressing these limitations in future research will significantly improve the applicability, robustness, and clinical relevance of the proposed methods.

### 4.4. Comparison with Existing Studies

Table 7 shows a comparison of the method proposed in this paper with other related studies on the same dataset. It can be seen that previous works vary in their emphases on feature extraction and classification model selection. For instance, they have incorporated multiple time-frequency domain features (such as PSD, power features in various frequency bands, and nonlinear complexity measures) and employed a range of machine learning algorithms, including SVM, DT, NB, and LDA. Overall, the existing research has reported accuracies ranging from 88% to 95%, mostly relying on carefully selected feature sets and relatively well-established classifiers.

In contrast, the approach proposed in this paper takes feature engineering a step further. In addition to conventional linear features, the brain areas are segmented (e.g., distinguishing left and right temporal lobes), and nonlinear features are incorporated to enhance the capture of subtle EEG signal differences. Moreover, for model design, a stacking strategy is adopted to integrate KNN, DT, and XGBoost models in a hierarchical fashion. This multi-model ensemble approach helps leverage the strengths of each classifier and improves the recognition accuracy of complex EEG data.

From the table, it is evident that the proposed method achieves a highest accuracy of 98.07% on the same dataset, which is notably superior to the results reported by other publications. This finding suggests that by appropriately selecting brain regions and combining both linear and nonlinear features—as well as by using a more targeted ensemble classification strategy—it is possible to extract more EEG information related to emotional and cognitive states, thereby significantly boosting classification performance.

## 5. Conclusions

This study utilized machine learning methods to investigate the effects of depression on different brain regions. The results showed that the stacking model achieved the highest detection accuracy of 98.07% when applied to the left temporal lobe region. Following closely were the KNN, SVM, and NN models, which achieved high accuracy rates of 96.97%, 96.64%, and 96.64%, respectively, with AUC values of 99.71%, 99.2%, and 99.81%. The experimental results indicated that patients with depression exhibit lower relative power in the Alpha and Gamma bands and higher relative power in the Beta band across most brain regions. The differences in EEG signals were particularly pronounced in the left and right temporal lobes, confirming the critical role of the temporal lobes in EEG-based depression diagnosis. Specifically, feature extraction from the left temporal lobe significantly contributed to classification performance. This study achieved high classification accuracy even with data from fewer brain regions, demonstrating the ability to simplify the detection process while retaining sufficient diagnostic capability. Compared to studies relying on data from a greater number of channels, this approach showed greater efficiency and provides theoretical support for the design of portable EEG devices in the future.

## Figures and Tables

**Table 1:** Brain region electrode mapping.

	Brain Region	Channels
1	Frontal	Fp1, Fp2, F3, F4
2	Left Temporal	F7, T3, T5
3	Parietal–Occipital	P3, P4, O1, O2
4	Right Temporal	F8, T4, T6
5	Central	C3, C4, Fz, Cz, Pz

**Table 2:** List of features included.

Feature Category	Feature Name	Feature Description	Feature Count
Spectral Features	Center Freq Relative	Relative center frequency, calculated by Simpson integration of the frequency multiplied by PSD.	5 regions × 9 features = 45
	Center Freq Absolute	Absolute center frequency; the frequency corresponding to the maximum value of PSD.	
	Delta Power	Power in the Delta band (1–3 Hz), calculated by Simpson integration of the PSD in this band.	
	Theta Power	Power in the Theta band (4–7 Hz).	
	Alpha Power	Power in the Alpha band (8–11 Hz).	
	Beta1 Power	Power in the Beta1 band (12–20 Hz).	
	Beta2 Power	Power in the Beta2 band (21–29 Hz).	
	Gamma Power	Power in the Gamma band (30–48 Hz).	
	Total Power	Total power, calculated by Simpson integration of the PSD across the entire frequency range.	
Temporal Features	Skewness	Skewness of the signal, reflecting the symmetry of the signal distribution.	5 regions × 3 features = 15
	Kurtosis	Kurtosis of the signal, reflecting the sharpness of the signal distribution.	
	Peak Value	Maximum value of the signal.	
Nonlinear Features	Sample Entropy	Sample entropy, reflecting the complexity of the signal.	5 regions × 5 features = 25
	Higuchi FD	Higuchi fractal dimension, reflecting the fractal characteristics of the signal.	
	Hurst Exponent	Hurst exponent, reflecting the long-term dependency of the signal.	
	Shannon Entropy	Shannon entropy, reflecting the entropy value of the signal.	
	C0 Complexity	C0 complexity of the signal; the maximum amplitude obtained through FFT.	
Time-Frequency Features	Mean TF Energy	Mean energy calculated across all frequencies and time windows, reflecting the average energy level in the time-frequency domain.	5 regions × 2 features = 10
	Max TF Energy	Maximum energy calculated across all frequencies and time windows, reflecting the peak energy level in the time-frequency domain.	
**Total**			**95**

**Table 3:** Overview of classifiers.

Classifier Name	Function/Algorithm Principle	Characteristics
DT	Simulates a decision-making process for classification tasks.	Easy to understand and implement, low computational complexity, robust to missing data and irrelevant features.
KNN	Classifies by measuring the similarity between samples in the feature space and making a decision based on the majority vote of the K-nearest neighbors.	Simple and intuitive, no training phase required, suitable for small datasets.
RF	Combines multiple decision trees for classification or regression, using a voting mechanism to enhance accuracy.	High accuracy, capable of handling high-dimensional data, resistant to overfitting.
SVM	Employs a maximum-margin principle for classification and extends to nonlinear problems using an RBF kernel function.	Well suited for high-dimensional data, performs well on small sample datasets.
LGBM	A gradient boosting framework that utilizes single-side sampling and exclusive feature bundling techniques to improve speed and efficiency.	Efficient for large-scale datasets, with high speed and memory efficiency.
XGBoost	An optimized gradient boosting algorithm that integrates parallel processing and regularization techniques to control model complexity.	Automatically parallelizes data processing, reduces overfitting, fast training time.
GB	Enhances classification and regression performance by iteratively combining multiple weak learners to minimize error.	High predictive accuracy, particularly effective for complex datasets.
NN	Mimics the connectivity of neurons in the human brain; well suited for complex pattern recognition tasks.	Capable of processing complex nonlinear data, adaptive learning capabilities, ideal for high-dimensional data such as images and speech.

**Table 4:** Evaluation metrics of eight models.

Classification Models	Accuracy	Recall	F1 Score	ROC AUC
DT	91.95%	92.62%	91.97%	91.97%
KNN	96.97%	95.31%	96.91%	99.81%
RF	95.96%	95.29%	95.92%	99.40%
SVM	96.64%	95.29%	96.57%	99.71%
LGBM	94.63%	94.57%	94.58%	98.87%
XGBoost	93.29%	93.93%	93.31%	98.38%
GB	92.28%	95.33%	92.52%	95.73%
NN	96.64%	95.95%	96.61%	99.20%
Ours	98.07%	97.27%	98.16%	98.06%

**Table 5:** Best parameters of each model.

Classification Models	Best Parameters
DT	‘max_depth’: 20, ‘min_samples_split’: 10
KNN	‘n_neighbors’: 5, ‘weights’: ’distance’
RF	‘max_depth’: 10, ‘n_estimators’: 200
SVM	‘C’: 1, ’gamma’: ‘auto’, ’kernel’: ’rbf’
LGBM	‘learning_rate’: 0.1, ‘n_estimators’: 500
XGBoost	‘learning_rate’: 0.05, ‘n_estimators’: 1000
GB	‘learning_rate’: 0.1, ‘n_estimators’: 200
NN	‘hidden_layer_sizes’: (50, 50), ‘max_iter’: 1000, ‘solver’: ‘adam’
Ours	‘dt_max_depth’: 5, ‘knn_n_neighbors’: 5, ‘xgb_learning_rate’: 0.01, ‘xgb_max_depth’: 3, ‘xgb_n_estimators’: 100

**Table 6:** Detection performance of individual brain regions using the hybrid model.

Brain Region	Accuracy	Recall	F1 Score	ROC AUC
Frontal	87.61%	84.57%	87.38%	88.73%
Left Temporal	91.92%	90.62%	91.77%	93.24%
Right Temporal	85.28%	86.67%	85.51%	89.90%
Parietal–Occipital	87.94%	87.98%	87.93%	89.83%
Central	89.64%	88.57%	89.56%	91.20%

**Table 7:** Comparison with existing studies on the same dataset.

Study	Method	Classification Method	Best Accuracy
Yang, Jianli, et al. (2023) [12]	PSD + LZC + brain region combination (frontal, temporal, central)	SVM	92.4% (SVM)
Yang, Jianli, et al. (2025) [25]	Fusion of LZC, SE, and KC features in the EO state and the PSD and SE features in the EC state	SVM	94.58% (SVM)
Mahato et al. (2020) [26]	Band power features (Delta, Theta, Alpha, Beta, Alpha1, Alpha2) and Theta asymmetry (average and paired)	SVM, Logistic Regression (LR), Naïve-Bayesian (NB), DT	88.33% (SVM)
Mahato et al. (2024) [16]	Nonlinear features (SampEn, DFA) along with EEG band power features	Linear Discriminant Analysis (LDA), NB, LR, DT, SVM, Bagging	95.23% (SVM)
OURS	Brain region segmentation into left and right temporal lobes, linear and nonlinear features	Stacking (KNN, DT, XGBoost)	98.07% (Stacking)

## Data Availability

The EEG data can be download at https://figshare.com/articles/dataset/EEG_Data_New-/4244171 (accessed on 24 July 2024).
